# Statistical analysis plan for an international, double-blind, randomized controlled clinical trial on the use of phantom motor execution as a treatment for phantom limb pain

**DOI:** 10.1186/s13063-021-05962-7

**Published:** 2022-02-13

**Authors:** Eva Lendaro, Eric J. Earley, Max Ortiz-Catalan

**Affiliations:** 1 Center for Bionics and Pain Research, Mölndal, Sweden; 2 Department of Electrical Engineering, Chalmers University of Technology, Gothenburg, Sweden; 3 Operational Area 3, Sahlgrenska University Hospital, Gothenburg, Sweden; 4 Department of Orthopaedics, Institute of Clinical Sciences, Sahlgrenska Academy, University of Gothenburg, Gothenburg, Sweden

## Abstract

**Background:**

Phantom limb pain (PLP) is a detrimental condition that can greatly diminish the quality of life. Purposeful control over the phantom limb activates the affected neural circuitry and leads to dissolution of the pathological relationship linking sensorimotor and pain processing (which gives rise to PLP). An international, double-blind, randomized controlled clinical trial (RCT) on the use of phantom motor execution (PME) as a treatment for PLP is currently undertaken, where PME is compared to an active placebo treatment, namely phantom motor imagery (PMI).

**Methods and design:**

Sixty-seven subjects suffering from PLP in upper or lower limbs are randomly assigned in 2:1 ratio to PME or PMI interventions respectively. Subjects allocated to either treatment receive 15 interventions where they are exposed to the same VR-AR environments using the same device. The only difference between interventions is whether phantom movements are performed (PME) or imagined (PMI).

**Results:**

The primary outcome of the study is to examine whether 15 sessions of PME can induce a greater PLP relief, compared to PMI. The secondary objectives are to examine whether 15 sessions of PME provide a greater improvement in different aspects related to PLP compared to PMI, such as pain duration, pain intensity as measured by other metrics, and the patient’s own impression about the effect of treatment. Long-term retention of treatment benefits will be assessed as change in all the variables (both primary and secondary) between baseline and follow-up timepoints (at 1, 3, and 6 months post-treatment).

**Conclusion:**

This manuscript serves as the formal statistical analysis plan (version 1.0) for the international, double-blind, randomized controlled clinical trial on the use of PME as a treatment for PLP. The statistical analysis plan was completed on 3 August 2021.

**Trial registration:**

ClinicalTrials.govNCT03112928. Registered on April 13, 2017

SAP version: version: 1.0, date: 2021/08/03

Protocol version: This document has been written based on information contained in the study protocol published in Lendaro et al. (BMJ Open 8:e021039, 2018), in July 2018.

SAP revisions: Not applicable

## Introduction

Phantom limb pain (PLP) is a subtype of post-amputation pain with prevalence between 17 and 88% by one meta-analysis study [1] and lifetime prevalence between 76 and 87% by another recent systematic review [2]. A wide range of treatment methods for the condition have been proposed over the years [3, 4]; however, reviews and surveys have repeatedly stressed that there is little evidence from appropriate randomized controlled trials (RCTs) to guide treatment choice, be it pharmacological or non-pharmacological [5–8]. Although experts’ recommendations seem to converge on a handful of approaches (including graded motor imagery, mirror therapy, and amitriptyline), they are based on limited evidence with mixed results [9]. Consequently, the optimal treatment for PLP remains a challenge to this day, in part undoubtedly due to the uncertainty on its pathophysiology [10].

Recent systematic reviews have also highlighted how the vast majority of RCTs do not meet the necessary criteria and present flawed design, conduct, analysis, and/or reporting [5, 8]. Low-quality RCTs make replicability and meta-analysis harder to achieve, limiting the extent of the evidence in support of treatment recommendations. Evidence-based tools such as the Standard Protocol Items: Recommendations for Interventional Trials (SPIRIT) [11] and Consolidated Standards of Reporting Trials (CONSORT) [12] guidelines have become instrumental in helping researchers to develop high-quality protocols and outcome reports respectively: these are great antidotes against compromised scientific evidence. Yet, the risk for selective reporting of outcome and analysis persists [13, 14]. To this end, the *Journal of the American Medical Association* (*JAMA*) published a statistical analysis plan (SAP) guidance document in 2017 containing a checklist of minimum items to include when reporting details of the statistical analysis of RCTs [15]. To ensure the much-needed high-quality evidence in the field of PLP research, not only protocols, but also SAPs should be published.

Phantom motor execution (PME) has been found to reduce PLP in case studies and in a single-arm clinical trial [16–19]. This therapy is currently investigated in an international, double-blind, randomized controlled clinical trial which aims at confirming the previous results [20]. The protocol of the RCT has been previously published [20], and the current article presents the detailed SAP adhering to the checklist provided by the *JAMA* guidelines [15]. Here, we describe the pre-specified statistical analysis principles and procedures to be followed by the statisticians responsible for analyzing the trial data.

## Statistical analysis plan

### Introduction and objectives

#### Synopsis of the trial background

PME promoted by myoelectric pattern recognition, virtual/augmented reality (VR/AR), and serious gaming is investigated in an international, double-blind, randomized controlled clinical trial [20], for which this SAP was created. Specifically, PME is compared to phantom motor imagery (PMI), which is implemented by keeping everything identical as in the experimental intervention (same device, VR/AR environments, and interaction with therapists), except that phantom movements are only imagined and not executed.

#### Study hypothesis

The working hypothesis of PME is that the purposeful activation of the affected motor circuitry will dissociate the pathological relationship between sensorimotor and pain processing resulting in PLP [19]. Because PMI does not engage as much of the sensorimotor neural circuitry as PME, the latter is hypothesized to be more likely to reduce PLP, framing the investigation as a superiority trial.

#### Study objectives

The primary objective of the study is to examine whether 15 sessions of PME can induce a greater PLP relief, compared to PMI. The secondary objectives are to examine whether 15 sessions of PME provide a greater improvement in different aspects related to PLP compared to PMI. Aspects considered by the secondary objectives are other measures of PLP such as pain duration, pain intensity as measured by other metrics, and the patient’s own impression about the effect of treatment. Another important aspect investigated in the trial is the long-term retention of treatment benefits, and therefore, another secondary objective is to assess whether PME brings a greater positive change in all the variables (both primary and secondary) between baseline and follow-up timepoints (1, 3, and 6 months post-treatment).

### Trial methods

#### Trial design

The trial protocol was previously published [20] and is briefly described here. Sixty-seven subjects with upper or lower limb amputations were planned to take part in this study. Subjects were assigned randomly to PME and PMI treatments (2:1 proportion). The study consists of baseline assessment, 15 treatment sessions of 2 h each, and three follow-up interviews at 1, 3, and 6 months post-treatment. The design is double-blinded as the patients were informed that the treatment received, regardless of which, has been shown effective in previous studies and were therefore unaware to be assigned to the active control arm. The evaluators did not take part in providing treatment (evaluator and therapist were different persons), making the study a double-blinded. In addition, the data analysis will be performed by a person different than the therapist, the evaluator, or the person that randomized the subjects.

#### Interventions

The experimental intervention consists of PME decoded via myoelectric pattern recognition and promoted via serious gaming in virtual and augmented reality. The active comparator consists of PMI in which the participants imagine performing the movements visualized in the virtual environments instead of executing them. In PMI, myoelectric activity is used to monitor that the subjects do not produce muscular contractions but only imagine the movements. Conversely, myoelectric activity is used to drive the actions taking place in the virtual environments in PME.

#### Randomization

Participants that meet the inclusion criteria (see protocol [20]) and signed the informed consent were assigned to the experimental or active control group in proportion 2:1, according to the optimal allocation scheme of minimization. Initial description and methods of minimization were introduced independently by Taves [21] in 1974 and Pocock and Simon [22] in 1975. In short, the minimization algorithm was chosen because it allows dynamic allocation of the subjects, in which each allocation is influenced by the current state of overall treatment balances. Specifically, the minimization schemes ensure that allocation balance is maintained by giving higher allocation probabilities to interventions selected in favor of reducing total imbalance. The randomization strategy was chosen to reduce the likelihood of baseline imbalance of potentially prognostic covariates between the two treatment groups and to maximize the amount of information collected on the experimental treatment. The following minimization factors were considered: level of amputation (upper and lower), baseline PLP based on the Numeric Rating Scale (NRS) (low 1–4 and high 5–10), and investigation site (eight centers). The minimization process is conducted using the opensource desktop application MinimPy [23] operated by the monitor of the clinical trial. Patients were randomized as they were enrolled. Specifically, a therapist at the investigation site was responsible for evaluating the eligibility of the subject by carrying out all the assessments included in visit 0. If the patient was deemed eligible, the therapist would assign a subject ID, which also identifies the investigation site, and communicate it to the monitor of the study together with information regarding the abovementioned minimization factors (level of amputation and the NRS value of PLP). The monitor would then input these values in the software and get as result the allocation group. The allocated treatment would then be communicated back to the therapist responsible for the treatment.

#### Sample size

This confirmatory clinical trial builds on the results of three previous studies [16–18]. Sample size calculation was informed by a previous one-armed clinical investigation of PME [17], where pain decrease in PRI was found to be 51% relative mean improvement with an absolute mean improvement of 9.6 (*SD* 8.1) and effect size of 1.18. In the one-armed trial, the PRI was computed using the pain descriptors as in the Short-Form McGill Pain Questionnaire [24] and scored individually using the present pain intensity scale [25] (scale none to excruciating, 0 to 5), thus giving a score with range 0 to 75. In the present study, we used the classic version of the Short-Form McGill Pain Questionnaire, which scores the single pain descriptors on a scale from 0 to 3 (none to severe), thus reducing the range to 0–45. The absolute mean improvement on the new scale would then become 5.75 and maintaining the same effect size yields an *SD* of 4.89. Considering that in this trial we had two groups and some improvement for the control group was expected, we chose a difference of 4 between the two groups (which would grant a reduction above 30%) to be adequate and still representative of a clinically meaningful change [26]. To find this difference between the two randomized groups (in proportion 1:2) in the primary outcome measure, with power of 80% resulting from a two-sided Fisher’s non-parametric permutation test at 5% significance level, at least 60 participants are required. Considering a drop-out rate of 10%, which is akin to what was found in the previous clinical investigation, at least 66 patients are expected to be randomized in total. Sample size calculation was carried out using SAS 9.2 PROC.

#### Framework

This trial uses a superiority hypothesis testing framework between treatment groups for all outcomes.

#### Timing of outcome assessments

See the trial protocol [18] and Table 8 in the Appendix for further details of all outcome measures and their assessment timings.

#### Statistical interim analysis and stopping guidance

There are no planned interim analyses for efficacy and no planned interim assessment for futility. The monitor of the study will provide stopping guidance should the necessity arise due to adverse events. The trial will be stopped after 60 subjects have completed the study.

#### Timing of final analysis

The final analyses and unblinding of the data will take place only after the database is locked, and SAP is signed. The first part containing primary analyses and all the data relative to the treatment (collected between visit 0 and visit 15) will be locked after all subjects have completed the treatment phase. The second part of the database containing all the data relative to the follow-up assessments will be locked after all subjects have completed the last follow-up visit.

### Statistical analysis principles

#### Confidence intervals and *p-*values

The main results will be presented as mean differences with 95% confidence intervals between the two groups together with effect sizes and *p*-values. All significance tests will be two-sided and conducted at the 5% level.

#### Multiple comparisons and multiplicity

For the confirmative analyses, the fixed sequential method will be used for controlling type I error, as indicated in Dmitrienko et al. [27]. This theoretical framework will be applied for primary analysis and important secondary analyses. If a test gives a significant result at the 5% significance level, the total test mass will be transferred to the following number in the test sequence until a non-significant result is achieved. All the significant tests before the first non-significant test will be considered confirmative. If the primary analysis will be non-significant, then no analysis will be confirmative.

#### Adherence and protocol deviations

A protocol violation in eligibility is defined as when a patient was randomized but did no longer qualify for the study according to the eligibility criteria (defined in the study protocol [20]). In addition, patients who withdrew their consent after randomization will be excluded from further follow-up and analysis. A protocol deviation from the assigned intervention can occur when a patient is assigned to either PME or PMI, but at any time during the study, the participant did not receive the prescribed intervention resulting in fewer than 15 sessions in total and/or gaps longer than two consecutively skipped sessions. Successful protocol adherence is defined as successfully completing the baseline assessment and the 15 treatment sessions without any protocol violation in eligibility, protocol deviation, and/or withdrawal of consent. Patients lost to follow-up are patients that completed the treatment without any major protocol violation or deviation but that did not complete the follow-up.

#### Analysis populations

- *The intent-to-treat (ITT) population:* The ITT population will be all patients randomized except patients withdrawing consent for the use of data or patients with a protocol violation concerning eligibility.
- The full analysis set (FAS): The full analysis set will be all subjects in the ITT population without imputation.
- *Per-protocol (PP) population:* All randomized subjects with no major protocol violations and deviations will be included in the per-protocol (PP) population.

**Fig. 1:**
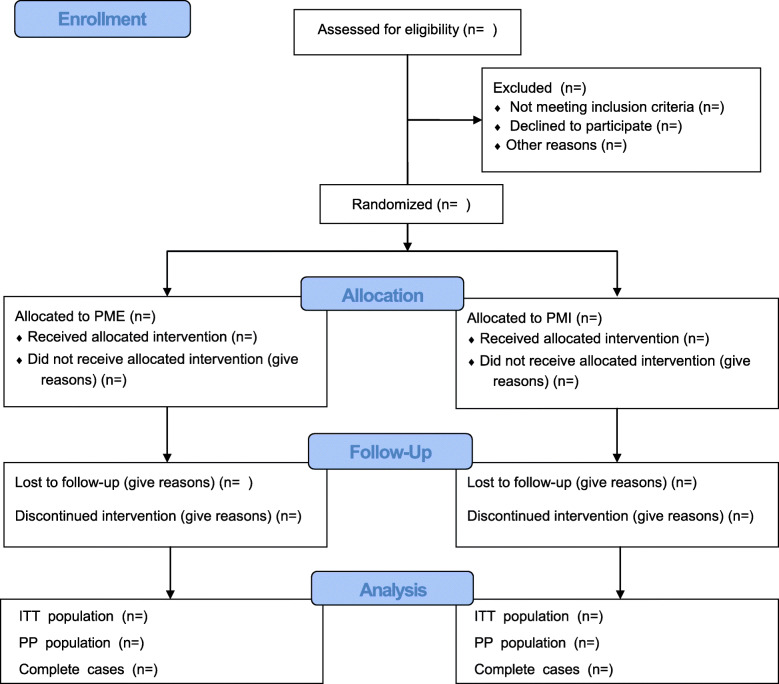
CONSORT flowchart. Abbreviations: PME phantom motor execution, PMI phantom motor imagery

Overall protocol adherence, violations, and deviations for the entire clinical trial will be summarized in the CONSORT flowchart presenting the patient inclusion for ITT and PP analyses (see Fig. 1 for the template). Detailed results of treatment allocation and adherence per investigational site will also be presented using Table 1 in the Appendix.

The ITT population, FAS, and the PP population will be specified in detail at the Clean file meeting before the database lock and before breaking the code. The number of subjects included in each of the ITT and PP populations will be summarized for each treatment group and overall. The number and percentage of subjects randomized and treated will be presented. Subjects who completed the study and subjects who withdrew from the study prematurely will also be presented with a breakdown of the reasons for withdrawal by treatment group for the ITT and PP populations.

### Trial population

#### Screening data and recruitment

We will report the number of screened patients who met study inclusion criteria, the number of excluded patients, and the number of participants included in the final analyses. The flow of trial participants will be displayed in a CONSORT diagram. The diagram will present the level of withdrawal (e.g., whether participants withdrew from intervention and/or from follow-up) and the timing and reasons of withdrawal/lost to follow-up data will be presented separately.

#### Baseline characteristics

A complete list of all the outcome measures assessed during baseline (visit 0) will be presented similarly to Table 8 in the Appendix, while the list of baseline variables will be reported similarly in Table 3 in the Appendix. Additionally, the level of amputation (whether it is an upper or lower limb amputation), investigational site, and level of PLP (high if ≥ 5, or low) were the minimization factors considered for the randomization. Categorical data will be summarized by numbers and percentages. Continuous data will be summarized by mean, standard deviation (SD), median, Q1, Q3, minimum, and maximum. Demographics and baseline characteristics will be summarized by treatment group for the ITT and PP populations. Tests of statistical significance will be performed for baseline characteristics but not included in the baseline tables in the article reporting the results; rather, the clinical importance of any imbalance will be noted.

#### Prior and concomitant medications

The use of concomitant medications is allowed provided that at the time of inclusion, the patient has stable consumption for at least 1 month before entering the study and any pain reduction potentially attributable to the drug occurred at least 3 months before entering the study. Medication intake is thus considered as a baseline variable but also monitored throughout the study as an efficacy variable. Prior and concomitant medication at baseline will be summarized by higher level Anatomical Therapeutic Classification (ATC) group and generic term for each treatment group for the ITT population.

#### Outcome variables

Table 8 in the Appendix summarizes the schedule of data collection for the trial.

##### Primary outcome variable

**Fig. 2:**
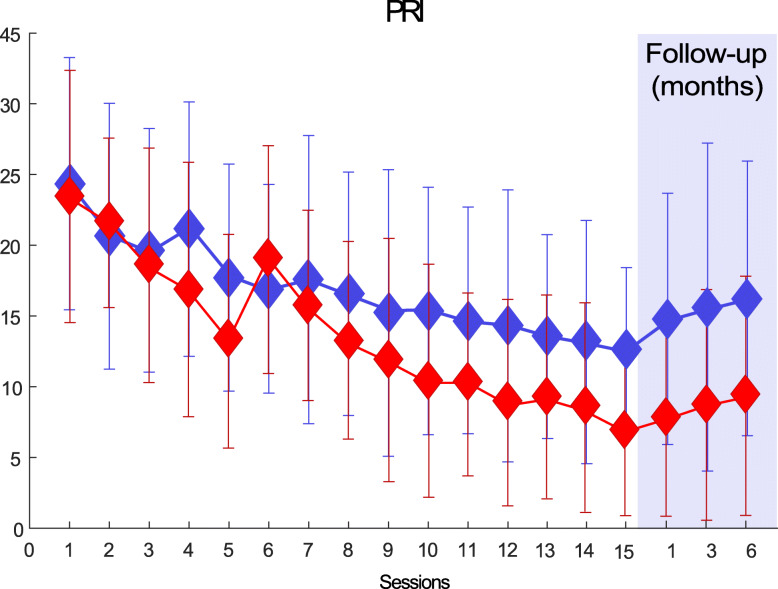
Example of subjects’ profile of Pain Rating Index (PRI) over time by treatment group. The data used for this plot are fictitious

The primary efficacy variable is the Pain Rating Index (PRI) (Fig. 2). The PRI is a continuous variable computed as the sum of the scores for all descriptors of the Short Form of the McGill Pain Questionnaire (SF-MPQ). The primary outcome of the study is the change in PRI between baseline (visit 0) and end of treatment (visit 15).

##### Secondary outcome variables

The secondary efficacy variables are other measures related to PLP and are listed below. As we are following the theory of fixed sequential multiple testing, the variables will be tested in the order in which they appear in the list below. All the variables apart from the Patients’ Global Impression of Change are assessed as changes between baseline and the specified timepoint.

- Patients’ Global Impression of Change measured at visit 15 (single score [1–7])
- PRI change between baseline and follow-up timepoints (1, 3, and 6 months)
- PRI defined as a dichotomous variable indicating whether the patient has experienced a clinically meaningful reduction in pain (achieved with a decrease 50% or more [28] of the PRI)
- From the Questionnaire for Phantom Limb Pain (Q-PLP):
  - Weighted Pain Distribution Index (range [1–5]): sum of the scores given to pain ratings during the day, weighted by the amount of time spent in the respective level of pain [16]
  - PLP intensity (NRS, [0–10])

Outcomes will be evaluated as changes between baseline and end of treatment and baseline and follow-up timepoints, if not otherwise stated.

##### Exploratory outcome variables

- EuroQol-5D-5L total score (mean value of the score given to the 5 items, [1–5])
- Pain Catastrophizing Scale Short Form total score (sum of the individual scores [0–24])
- Pain Disability Index (sum of the scores given to each item [0–70])
- Patient Health Questionnaire total score (sum of the individual scores [0–6])
- Pain Self-Efficacy Questionnaire total score (sum of the individual scores [0–12])
- Health Care Climate Questionnaire (HCCQ) total score (sum of the individual scores)
- Expectations for Complementary and Alternative Medicine Treatments Short Form (EXPECT-SF) total score
- Opinion About Treatment (OAT), total score [3–18]
- From the Questionnaire for Phantom Limb Pain (Q-PLP):
  - PLP intensity on Present Pain Intensity (PPI) scale, categorical, 6 levels
  - Telescoping (1 most proximal–6 most distal)
  - Pain locations (1 most proximal–6 most distal, multiple selection possible)
  - Stump pain, NRS, [0–10]
  - Medication consumption (improved or not)
  - Pain interference with work, NRS, [0–10]
  - Pain interference with daily life activities (NRS, [0–10])
  - Pain interference with sleep, NRS, [0–10]
  - Pain qualities from the McGill Questionnaire
  - Pain frequency (improved or not)
  - Exponential decay model of trial-by-trial improvement

Outcomes will be evaluated as changes between baseline and end of treatment and baseline and follow-up timepoints, if not otherwise stated.

##### Safety variables

Safety variables for the trial are treatment-emergent adverse events (that is, events either not present at baseline or present at baseline but of increased severity) and any serious adverse event (SAE).

All variables are measured at visit 0 (baseline), visit 15 (end of treatment), and follow-up assessments, if not otherwise specified. Adverse events will be reported as cumulative percentages at the end of the trial by treatment group.

### Analysis

#### General statistical methodology

All the main analyses will be performed on the ITT or FAS population between the two randomized groups. Complementary analyses will be performed on the PP population. For the confirmatory analyses, the fixed sequential test method for adjustment for multiplicity will be applied started with primary analysis and followed by the secondary analyses as numbered above. All other analyses will be exploratory. For comparison between the two randomized groups, Fisher’s non-parametric permutation test will be used for continuous variables, Fisher’s exact test for dichotomous variables, Mantel-Haenszel chi-square test for ordered categorical variables, and Pearson chi-square test for non-ordered categorical variables. Adjusted analyses will be performed with analysis of covariance for continuous outcome variables and with logistic regression for dichotomous outcome variables.

The main results will be the mean differences with 95% CI between the two groups, effect sizes, and *p*-values. For analyses of changes within groups, Fisher’s non-parametric permutation test for paired observation will be used for continuous variables and the Sign test for dichotomous and ordered categorical variables. All correlations will be performed with Spearman’s correlation coefficient.

All statistical tests will be two sided and conducted at the 5% significance level. The distribution of continuous variables will be given as mean, SD, median, Q1, Q3, minimum, and maximum, and distribution of categorical variables will be given as numbers and percentages. All major changes from the statistical analysis plan will be specified by subdividing into before and after database lock and unblinding. Normality of the data will be assessed inspecting the histograms.

#### Analysis of the primary outcome variable

The main analyses will be performed using the ITT population and it will be unadjusted. The comparison of change in PRI between baseline (visit 0) and end of treatment (visit 15) between the two treatment groups will be carried out with a two-sided Fisher’s non-parametric permutation test on significance level 0.05. We chose to use a non-parametric test for the main analysis as it does not rely on the assumption that the data are normally distributed, which we cannot ensure a priori. Missing data will be imputed with stochastic imputation using 50 datasets; it should be noted that this method of imputation differs from the use of last observation carried forward (LOCF) proposed in our previous work [20], as advised by our senior statistician consultant and reviewers. The difference in change in PRI with 95% CI will be given together with effect size and *p*-value.

*The following sensitivity analyses will be performed for the primary analysis:*

1. The robustness of the estimate of treatment effect will be assessed by adjusting for the baseline characteristics used for randomization (level of PLP at baseline (NRS), baseline PRI, level of amputation). For adjusted comparison between two groups, the analysis of covariance (ANCOVA) will be used with intervention/control as the independent variable and the baseline characteristics used for randomization as covariates. The effect estimates will be presented as group difference with 95% confidence intervals. This analysis will be conducted on the ITT population using stochastic imputation to handle missing data.
2. The impact of the imputation method will be assessed by running the same analysis as point 1 using LOCF for missing data imputation.
3. The impact of missing data will be assessed by running the unadjusted comparison on the full analysis set (FAS) using the same statistical methods as in primary analyses without imputing missing data.

A complementary analysis on the primary variable will also be performed on the PP population.Changes within groups will also be performed but with lower evidence value.

#### Analysis of secondary outcome variables

All secondary outcome variables will be analyzed between the two randomized groups according to the statistical methods presented in the section general statistical methodology, both on the ITT population using stochastic imputation and on the PP population. Within-group comparison will also be performed on the ITT population.

#### Analysis of exploratory outcomes

All exploratory outcome variables will be analyzed between the two randomized groups according to the statistical methods presented in the section general statistical methodology, on the ITT population using stochastic imputation. Within-group comparison will also be performed on the same population. Prosthetic usage data will be descriptively analyzed.

#### Subgroup analysis

The effect on the primary outcome will be analyzed also in subpopulations defined by: level of amputation, cause of amputation and gender. Subgroup analyses will be carried out using the approach followed for the main unadjusted comparison.

#### Missing data

The percentage and absolute withdrawal of participants lost to follow-up will be reported for each study arm in the CONSORT flowchart and reasons for missing data will be documented.

#### Safety analyses

Only treatment-emergent AEs will be included in the summaries for the safety population. The number and percentage of patients experiencing SAE will be presented for each treatment arm.

#### Software details

MATLAB version 9.10.0 (or above) will be used for all analyses [29].

## Conclusion

The present article describes the analysis principles and specific statistical procedures chosen for analyzing the primary, secondary, and exploratory outcomes of an international, double-blind, randomized controlled clinical trial on the use of phantom motor execution as a treatment for phantom limb pain [20]. The SAP has been developed in accordance with the *JAMA* guidelines [15] and the main objective is to make it publicly available before unblinding, in order to minimize the risk of outcome reporting bias.

The statisticians and clinical investigators who developed the plan were blinded to the treatment allocation and treatment-related study results and will remain blinded until the database is locked for final data extraction and analysis. The trial is registered at ClinicalTrials.gov with registration ID NCT03112928 and began recruiting patients in May 2017. The final follow-up visit is expected to be conducted in August 2021. The data is expected to be ready for analysis and unblinded in December 2021.

## Figures and Tables

**Table 11:**
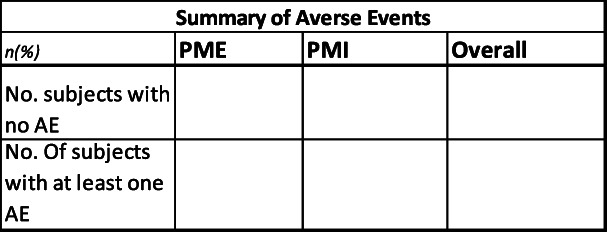
Summary of adverse events (ITT population)

*Abbreviations: PME* phantom motor execution, *PMI* phantom motor imagery

## Data Availability

Not applicable

## Appendix

Templates for tables and figures

**Table 1:** Subject disposition by treatment group

Subject disposition by treatment group			
*n(%)*	PME	PMI	Overall
Total screened subjects			
Subjects randomized			
Started treatment			
Completed Treatment			
Did not complete treatment			
Completed follow-up			
Protocol deviations/violations			
Lost to follow-up			
Safety population			
ITT population			
PP population			

*Abbreviations*: *ITT* intention to treat, *PP* per protocol, *PME* phantom motor execution, *PMI* phantom motor imagery

**Table 2:** Protocol deviations leading to exclusion from the PP population (ITT population)

Protocol deviations leading to exclusion from PP population (ITT population)			
*n(%)*	PME	PMI	Overall
Number of subject with at least on protocol deviation			
Number of subject with at least on protocol deviation			
Major protocol violations for subject excluded from the per protocol population			
Violation 1			
Violation 2			

*Abbreviations*: *PME* phantom motor execution, *PMI* phantom motor imagery

**Table 3:** Demographic and baseline characteristics for the ITT population

Baseline characteristics to be reported by treatment group (ITT population)					
*n(%) unless otherwise specified*			PME (n=)	PMI (n=)	Overall (n=)
***Background information***					
Age at randomization [years, mean (SD)]					
Sex [% male]					
Time since amputation [months, mean (SD)]					
Time since onset of PLP [months, mean (SD)]					
Reason of amputation	trauma				
	cardiovascular				
	cancer				
	other				
Type of prosthesis	none				
	active/myoelectric				
	cosmetic				
	passive				
	body-powered				
Daily prosthesis use [hours, mean (SD)]					
Minimization factors	Level of amputation	upper limb			
		lower limb			
	Intensity of PLP [NRS (0-10), mean(SD)]	high (> 4)			
		low (<= 4)			
	Investigational site	#1			
		#2			
		#3			
		#4			
		#5			
		#6			
		#7			
		#8			
		#9			
Chosen treatment frequency	once a week				
	twice a week				
	five times a week				
***Q-PLP***					
Intensity of stump pain [range, mean (SD)]					
Intensity of PLS [range, mean (SD)]					
Intensity of voluntary phantom movements [range, mean (SD)]					
PRI at baseline range, mean (SD)]					
Pain interference with sleep [range, mean (SD)]:					
Pain interference with daily life activities [range, mean (SD)]:					
Pain interference with work [range, mean (SD)]:					
Presence of telescoping [range, mean (SD)]					
Weighted Pain Distribution Index [range, mean (SD)]:					
***Other questionnaires***					
PDI [range, mean (SD)]:					
EQ5D-5L [range, mean (SD)]:					
PSEQ-2 [range, mean (SD)]:					
PCS-SF [range, mean (SD)]:					
PHQ-2 [range, mean (SD)]:					
EXPECT-SF [range, mean (SD)]:					

*Abbreviations*: *PME* phantom motor execution, *PMI* phantom motor imagery, *PLP* phantom limb pain, *NRS* Numeric Rate Scale, *Q-PLP* Questionnaire for Phantom Limb Pain, *PDI* Pain Disability Index, *PSEQ-2* 2-item Pain Self-Efficacy Questionnaire, *EQ-5D-5L* Euroqol-5D-5L, *PCS-SF* Pain Catastrophizing Scale Short Form, *PHQ-2* 2-item Patient Health Questionnaire, *PGIC* Patients’ Global Impression of Change, *OAT* Opinion About Treatment, *HCCQ* Health Care Climate Questionnaire, *EXPECT-SF* Expectations for Complementary and Alternative Medicine Treatments Short Form

**Table 4:** Demographic and baseline characteristics for PP population

Baseline characteristics to be reported by treatment group (PP population)					
*n(%) unless otherwise specified*			PME (n=)	PMI (n=)	Overall (n=)
***Background information***					
Age at randomization [years, mean (SD)]					
Sex [% male]					
Time since amputation [months, mean (SD)]					
Time since onset of PLP [months, mean (SD)]					
Reason of amputation	trauma				
	cardiovascular				
	cancer				
	other				
Type of prosthesis	none				
	active/myoelectric				
	cosmetic				
	passive				
	body-powered				
Daily prosthesis use [hours, mean (SD)]					
Minimization factors	Level of amputation	upper limb			
		lower limb			
	Intensity of PLP [NRS (0-10), mean(SD)]	high (> 4)			
		low (<= 4)			
	Investigational site	#1			
		#2			
		#3			
		#4			
		#5			
		#6			
		#7			
		#8			
		#9			
Chosen treatment frequency	once a week				
	twice a week				
	five times a week				
***Q-PLP***					
Intensity of stump pain [range, mean (SD)]					
Intensity of PLS [range, mean (SD)]					
Intensity of voluntary phantom movements [range, mean (SD)]					
PRI at baseline range, mean (SD)]					
Pain interference with sleep [range, mean (SD)]:					
Pain interference with daily life activities [range, mean (SD)]:					
Pain interference with work [range, mean (SD)]:					
Presence of telescoping [range, mean (SD)]					
Weighted Pain Distribution Index [range, mean (SD)]:					
***Other questionnaires***					
PDI [range, mean (SD)]:					
EQ5D-5L [range, mean (SD)]:					
PSEQ-2 [range, mean (SD)]:					
PCS-SF [range, mean (SD)]:					
PHQ-2 [range, mean (SD)]:					
EXPECT-SF [range, mean (SD)]:					

*Abbreviations*: *PME* Phantom Motor Execution, *PMI* Phantom Motor Imagery, *PLP* Phantom Limb Pain, *NRS* Numeric Rate Scale, *Q-PLP* Questionnaire for Phantom Limb Pain, *PDI* Pain Disability Index, *PSEQ-2* 2-item Pain Self-Efficacy Questionnaire, *EQ-5D-5L* Euroqol-5D-5L, *PCS-SF* Pain Catastrophizing Scale Short Form, *PHQ-2* 2-item Patient Health Questionnaire, *PGIC* Patients' Global Impression of Change, *OAT* Opinion About Treatment, *HCCQ* Health Care Climate Questionnaire and *EXPECT-SF* Expectations for Complementary and Alternative Medicine Treatments Short Form

**Table 5:** Prior medications

n(%)		Patients with prior use of drug	
***ATC group***	***Generic term***	***PME***	***PMI***
*example*			
*ATC group 1*	*Drug 1*		
*ATC group 2*	*Drug 2*		

*Abbreviations*: *PME* phantom motor execution, *PMI* phantom motor imagery

**Table 6:** Concomitant medications

n(%)		Patients using drug		Case drug discontinued (during study)	
***ATC group***	***Generic term***	***PME***	***PMI***	***PME***	***PMI***
*example*					
*ATC group 1*	*Drug 1*				
*ATC group 2*	*Drug 2*				

*Abbreviations*: *ATC* Anatomical Therapeutic Chemical group, *PME* phantom motor execution, *PMI* phantom motor imagery

**Table 7:** Prior treatment

n(%)	Patients with prior treatment	
***Treatment type***	***PME***	***PMI***
*Treatment 1*		
*Treatment 2*		

*Abbreviations*: *PME* phantom motor execution, *PMI* phantom motor imagery

**Table 8:** Summary of the outcome assessment schedule

Session	Summary of content
Visit 0	• Patient information (T/E) • Study consent (T/E) • Pre-assessment (T/E) • Background information (T/E) • Q-PLP (T/E) • PDI (T/E) • EQ-5D-5L (T/E) • PSEQ-2 (T/E) • PCS-SF (T/E) • PHQ-2 (T/E) • EXPECT-SF (T/E)
Randomization	
Visit 1	• Treatment session (T) • Q-PLP (E) • OAT (E) • EXPECT-SF (E) • HCCQ-SF (E)
Visit 2–14	• Treatment session (T) • Q-PLP (E)
Visit 15	• Treatment session (T) • Q-PLP (E) • PDI (E) • EQ-5D-5L (E) • PSEQ-2 (E) • PCS-SF (E) • PHQ-2 (E) • PGIC (E) • HCCQ-SF (E)
1-month follow-up	• Q-PLP (E) • PDI (E) • EQ-5D-5L (E) • PSEQ-2 (E) • PCS-SF (E) • PHQ-2 (E)
3-month follow-up	
6-month follow-up	

*Abbreviations: Q-PLP* Questionnaire for Phantom Limb Pain, *PDI* Pain Disability Index, *PSEQ-2* 2-item Pain Self-Efficacy Questionnaire, *EQ-5D-5L* Euroqol-5D-5L, *PCS-SF* Pain Catastrophizing Scale Short Form, *PHQ-2* 2-item Patient Health Questionnaire, *PGIC* Patients’ Global Impression of Change, *OAT* Opinion About Treatment, *HCCQ* Health Care Climate Questionnaire, *EXPECT-SF* Expectations for Complementary and Alternative Medicine Treatments Short Form

**Table 9:** Primary efficacy analyses

Mean changes in primary efficacy outcome and group difference						
Analysis	Mean changes from baseline		Between group differences			
	PME	PMI	PME vs PMI	Treatment Effect (95% CI)		*p*-value
Main unadjusted comparison (ITT)						
Main unadjusted comparison (PP)						
Sensitivity analyses						

*Abbreviations*: *PME* phantom motor execution, *PMI* phantom motor imagery, *CI* confidence interval

**Table 10:** Summary of adverse events (ITT population)

Averse events			
*n(%)*	PME	PMI	Overall
Subjects with no AE			
Subjects with at least one AE			

*Abbreviations*: *PME* phantom motor execution, *PMI* phantom motor imagery

## Abbreviations

AEs: Adverse events
AR: Augmented reality
CONSORT: Consolidated Standards of Reporting Trials
FAS: Full analysis set
ATC: Anatomical Therapeutic Chemical (classification group)
ITT: Intent-to-treat
LOCF: Last observation carried forward
NRS: Numeric Rating Scale
PME: Phantom motor execution
PMI: Phantom motor imagery
PLP: Phantom limb pain
PP: Per protocol
PRI: Pain Rating Index
Q-PLP: Questionnaire for Phantom Limb Pain
SAP: Statistical analysis plan
SPIRIT: Standard Protocol Items: Recommendations for Interventional Trials
VR: Virtual reality
